# Notes and News

**Published:** 1902-12-20

**Authors:** 


### Notes and News.

Two new operating theatres have recently been presented to the Liverpool Infirmary by an anonymous donor.

The formation of an English committee to memorialise the late Professor Virchow has caused much gratification in Germany. The scheme of which Dr. Felix Semon is honorary secretary, is meeting with much success.

The guardians of the West Derby, Liverpool, and Toxeth Park Unions were the first to institute a Consumption Sanatorium for Poor Law patients, and their institute was opened in October. It was stated that the Bradford Sanatorium was the pioneer, but this is not correct.

A new wing has been opened at the Kettering General Hospital. The extension includes a ward for women, and the old ward for women will be utilised for children. The men's ward has been enlarged. Two small isolation wards have been added, also numerous offices, and additions have been made to existing departments. The outlay will amount to about £8,000.

A good deal of activity is visible amongst the management of the Radcliffe Infirmary, Oxford. There is now visible a desire to bring the institution up to modern requirements, and with this desire more interest in the work of the infirmary appears also to have grown up. The master of University College has promised a Finsen ray apparatus, and it is to be hoped that other friends will be found ready to remedy the defects and supply the necessary improvements pointed out in the well-arranged and intelligently compiled appeal now issued by the committee.

An international congress for the prevention of industrial diseases has been organised to take place at Milan in 1904, when an exhibition of industrial and professional hygiene will be held.

The post of Professor Superintendent of the Brown Animal Sanitary Institution is vacant. The salary is £250. Information can be obtained from the secretary, 149 Wandsworth Road, S.W.

The War Office is arranging to secure hospital accommodation for about 20,000 sick and wounded during times of war. It is proposed to utilise existing buildings, and act in co operation with municipal authorities.

The Berlin Municipality have decided to place a ward in the Municipal House of Refuge, at the disposal of patients who cannot immediately gain admission into the hospitals, and whose case is urgent. The accommodation will be for 65 patients.

American manufactures are finding favour in the matter of ambulances as elsewhere. Sir Vincent Kennett Barrington, of the Asylums Board, when visiting New York, expressed himself as struck with the superior rapidity of the American ambulances, and in Edinburgh an ambulance has been imported from Philadelphia.

Major Ronal Ross is the Englishman to whom on the present occasion the Nobel Prize for Medicine has been awarded. The Liverpool School of Tropical Medicine will benefit by the honour, which will reflect credit upon the choice of Major Ross as their pioneer in the study of malarial diseases. The King of Sweden presents the honour to the recipients personally.

The Examination Board of the Royal Colleges of Physicians and Surgeons of England having decided to hold examinations of provincial students in the large provincial centres of medical education, the test examination, Part V., Chemistry and Physics, and Part III., Elementary Biology, and the Preliminary Science Examination for the license in Dental Surgery, will be held at Owens College, Manchester, as well as in London.

The "Special Appeal Supplement" we issued last week, containing accurate and up-to-date facts, has attained the maximum of circulation. The accounts of each hospital, covering the whole field of its work and wants, has attracted the attention of all hospital supporters and all interested in their efficient maintenance. The Prince of Wales, we understand, was very much interested in reading it, and he regards it as most useful and handy for reference, owing to the full account it contains of the different hospitals. Lord Lister, too, has found this Supplement invaluable for the same reasons. It may be well to emphasise the fact that the more intelligent and liberal supporters of the hospitals attach value to the advertisements, because they state the requirements of each institution from the managers' point of view, and they are glad of the opportunity of comparing them with the particulars given in the editorial matter.

